# The impact of mobile Internet use on mental distress among Chinese adults during the COVID-19 pandemic

**DOI:** 10.3389/fpubh.2022.966606

**Published:** 2022-10-19

**Authors:** Mingming Teng

**Affiliations:** School of Economics and Trade, Guangdong Mechanical & Electrical Polytechnic, Guangzhou, China

**Keywords:** mobile Internet use, mental distress, COVID-19 pandemic, regression, mechanism analysis

## Abstract

With the rapid development of digital technology, mobile Internet use is increasing in popularity in China. Previous studies have shown that mobile Internet use has a positive or negative effect on mental distress. Using CFPS2020 data, this paper finds that mobile Internet use significantly alleviates mental distress in Chinese adults. Heterogeneity analysis indicates that mobile Internet use can significantly alleviate mental distress among adults between the ages of 30 and 70, without a bachelor's degree or residing outside the province of Hubei. Furthermore, mobile Internet use significantly reduces mental distress through two mediators: trust and happiness. It also shows that watching short videos or learning online is associated with reduced mental distress, as opposed to online shopping, chatting, or playing games. However, the mental distress of new mobile Internet users in 2020 has not been alleviated. This paper enriches the relevant theoretical research and provides a practical reference for using the mobile Internet to ease mental distress during epidemics.

## Introduction

Anxiety or depression is a common mental disorder worldwide. According to the World Health Organization, about 3.8% of the global population is affected, including 5% of adults. The 2012–2015 China Mental Health Survey showed the lifetime prevalence of depression among Chinese adults reached 6.8% and its adequate treatment rate was only 0.5% (1). As the Chinese digital economy develops, mobile phone penetration and Internet penetration are increasing (2, 3). Online chatting, shopping, learning, and playing games are becoming integral parts of people's daily lives (4, 5).

In previous studies, there has been no consistent conclusion regarding the impact of Internet use on mental health. Studies have found that excessive Internet use may result in Internet addiction and depression (6, 7). As a result, young people become unmotivated in studying or even engaged in fraud (8–10). Other studies have indicated that Internet use can reduce anxiety and depression. Cotten et al. studied old adults in assisted and independent living communities in Alabama and found that internet use increased older adults' communication with others, which alleviated their loneliness (11). Zhang et al. conducted an analysis of CFPS data in 2016 and 2018 using the DID method and found that the use of the Internet significantly reduced depression levels (12). Adama and Alhassan found that mobile phone penetration can significantly improve the quality of life of individuals in 114 countries (13).

The COVID-19 pandemic not only poses a serious threat to people's physical health but also to their mental health (14, 15). Li et al. conducted an online survey of Chinese adults using the Generalized Anxiety Disorder-7 (GAD-7) and the Patient Health Questionnaire-9 (PHQ-9) to assess anxiety and depression severity. They found that COVID-19 was associated with a significant increase in anxiety and depression among Chinese adults (16). The rapid spread of COVID-19 can also cause vicarious traumatization to the public and medical personnel (17). Many governments have taken various public health emergency interventions to prevent the spread of COVID-19 (18). This may have resulted in a reduction in face-to-face communication between people, thus worsening loneliness, particularly among elderly people (19). As a result, how to effectively relieve people's mental distress under COVID-19 becomes of concern.

The previous literature mainly focuses on the relationship between Internet use and mental health, or the relationship between COVID-19 and mental health. Few studies have examined the relationship between Internet use and mental health during the COVID-19 pandemic. Duan et al. distributed questionnaires on Questionnaire Star to Chinese students in Hubei province, finding that students were suffering from smartphone addiction, Internet addiction, and worsening depression (20). Similarly, this study and Duan's study focus on the impact of Chinese Internet usage on mental health under COVID-19. However, they differ significantly in three respects. First, Li just focuses only on the Hubei Province in China. This study examines respondents from 32 provinces in China. Second, Li's research focuses on Internet addiction and overuse, while this study mainly examines the normal use of mobile Internet. Third, this paper takes into account the intermediary mechanism of trust and happiness.

This paper attempts to answer a new question: “Can mobile Internet use alleviate people's anxiety or depression during COVID-19?” We test the results of the basic regression using 2 Stage Least Square (2SLS) and Generalized Method of Moments (GMM); analyze heterogeneity among individuals of different ages, education levels, and regions; select two mediating variables for mechanism analysis; and conduct further research on Internet use patterns. This study contributes to the study of mental distress during COVID-19 and makes some useful recommendations, which have both theoretical and practical implications.

## Hypotheses

Since the outbreak of COVID-19, many countries have implemented lockdown measures in order to prevent the spread of the virus (21, 22). These measures have resulted in a reduction in interpersonal communication, thus contributing to loneliness or depression (23). The COVID-19 epidemic can aggravate mental distress all over the world (24, 25).

Previous literature found that Internet use can relieve anxiety. Wang et al. examined the 2016 CFPS data using propensity score matching and logistic regression models and found that older adults who were online had relatively lower levels of depression (26). Based on data from the US Health and Retirement Study (HRS) of older adults, Heo et al. found that Internet use could enhance life satisfaction (27). Internet access can facilitate communication with the outside world (28). Mobile Internet use can moderately relieve anxiety or depression if it is not overused. Meanwhile, the spread of COVID-19 can exacerbate people's anxiety or depression. Thus, we propose Hypothesis 1 (H1):

H1: Mobile Internet use can alleviate people's anxiety or depression during the COVID-19 epidemic.

With the rapid development of internet technology and instant messaging, people are increasingly interacting online. Before COVID-19, people's interpersonal relationships and trust were relatively fragile online (29). After COVID-19, people rely more on mobile phone internet for establishing connections and exchanging information (30). During the pandemic, people may increase their trust in each other through online communication, thereby alleviating depression.

People's fear of COVID-19 can lead to a reduction in happiness and an increase in mental pressure (31). Gong et al. found that through E-chat, adults were able to increase their flexibility and happiness during the epidemic (32). Matthes et al. found that Austrians used their smartphones to disclose themselves online, increase the amount of communication with others, and thus increase their happiness during the epidemic (33).

Thus, we propose Hypothesis 2 and Hypothesis 3 (H2 and H3) as follows:

H2: Mobile Internet use can alleviate mental distress by increasing people's trust.
H3: Mobile Internet use can alleviate mental distress by improving people's happiness.

## Data and methods

### Data collection

The data used in this paper were from the China Family Panel Studies (CFPS) database in 2020 (http://www.isss.pku.edu.cn/cfps/). It is a major project funded by Peking University and the National Natural Science Foundation of China (NSFC), which is being implemented by the China Social Science Survey (ISSS) of Peking University (34). This survey has been conducted every two years since 2010, in order to reflect changes in China's social, economic, educational, and health conditions. Since Coronavirus is widespread and lockdown policies are enforced, the 2020 CFPS survey was conducted mostly *via* telephone. There were 88.6% of telephone surveys conducted in 2020, compared to 21.1% in 2018.

The purpose of this study was to examine the impact of mobile Internet use on mental distress among Chinese adults. After data cleaning, we obtained a sample of 16,004 observations based on the CFPS 2020 survey data.

### Variables measurement

#### Dependent variable: Mental distress

Generally, mental distress is measured by the CES-D8 or the PHQ-4 (35), which indicates feelings of anxiety or depression. The ISSS used a condensed version of the CES-D8 to measure mental distress in the CFPS2020 survey. In order to compare depression scores between 2012, 2014, and 2016, the survey organization used equipercentile equating the scores from the CES-D8 and CES-D20 sets to generate the CES-D20 for 2020. Consequently, this study adopted the ISSS's recommendation to use CES-D8 for measuring individual depression levels, and use CES-D20 as an alternative dependent variable for robustness. CES-D8 scores range from 8 to 32 on the CFPS2020 questionnaire. Those with a score of 16 or greater were considered to be depressed.

#### Independent variable: Mobile Internet use

This variable is derived from question number U201 of the CFPS 2020 questionnaire, which asks, “Do you use a mobile device, such as a mobile phone or tablet PC, to access the Internet?” If the answer is yes, then the variable is 1, otherwise, it is 0.

#### Instrumental variables: Mobile penetration and county average

We selected two instrumental variables to mitigate the potential endogenous problems. The variable Mobile Penetration is the mobile phone penetration rate in each individual's city, which is derived from the cell phone penetration rate in the China City Statistical Yearbook. The variable County average is the average time (minutes) spent on the Mobile Internet in an individual's county. It is calculated based on the CFPS survey question, “How long do you spend on the Internet on your mobile device (in minutes).”

#### Mediating variables: Trust and happiness

The mediating variables are Trust and Happiness. The variable Trust is assessed by asking the question, “In general, do you believe that most people are trustworthy, or should you take greater precautions when dealing with others?” (Question No. N1001). When the answer is that most people are trustworthy, the variable is set to 1, otherwise, it is set to 0. The variable Happiness is from the question “How happy do you think you are” (Question No.M2016). Individuals can rate their level of happiness on a scale of 0–10, with 10 representing the highest level of happiness and 0 representing the lowest level of happiness.

#### Control variables

According to previous related studies (36), this study selected variables related to individual characteristics as control variables. They are age (18–95 years old), gender (1 = male, 0 = female), marital status (1 = Married or having a spouse, 0 = others), employment (1 = Employmented, 0 = others), education (0 = Illiterate/Semi-literate, 3 = Primary school, 4 = Junior high school, 5 = Senior high school, 6 = 3- year college, 7 = Bachelor's program, 8 = Master's program, 9 = Doctoral program), insurance (1 = have any of the following medical insurances, e.g., Public medical insurance, Urban Employment Basic Medical Insurance, Urban Resident Basic Medical Insurance, Supplementary medical insurance, New Rural Cooperative Medical Insurance, 0 = others). And we also controlled the provincial dummy variable.

Table 1 shows the descriptive statistics of the main variables of this paper. The range of CES-D8 scores in the observations is 8–32, while the range of CES-D20 scores is 22–72. The higher the score, the greater the individual's level of depression or anxiety. The mean VIF of the main variables is only 1.32, which is far below the threshold of 10, indicating that there is no significant multicollinearity.

**Table 1:** Descriptive statistics.

**Variables**	***N***	**Mean**	**SD**	**Min**	**Max**
CES-D8	16,004	13.55	4.112	8	32
CES-D20	16,004	33.18	8.162	22	72
Mobile Internet use	16,004	0.628	0.483	0	1
Gender	16,004	0.495	0.500	0	1
Education	16,004	3.701	2.035	0	10
Age	16,004	47.53	16.01	18	95
Marital status	16,004	0.819	0.385	0	1
Employment	16,004	0.721	0.449	0	1
Insurance	16,004	0.892	0.310	0	1
Mobile Penetration	16,004	0.0117	0.00331	0.00735	0.0241
County average hours	16,004	98.91	37.48	19.23	258.9
Trust	15,936	0.595	0.491	0	1
Happiness	15,996	7.492	2.084	0	1

### Model design and data analysis

#### The effect of mobile Internet use on mental mistress

First, we used OLS to estimate the impact of mobile Internet use on mental distress during COVID-19. The basic regression model in this paper is:


(1) $$\begin{array}{l} \text{Menta}{\text{l}}_{\text{i}}=\alpha_{0}+\alpha_{1}\text{Mobil}{\text{e}}_{\text{i}}+\alpha_{2}Z_{\text{i}}+\alpha_{3}province+\varepsilon \end{array}$$

Where Mental_i_ represents the mental distress of individual i, which is the level of depression measured by CES-D8, Mobile_i_ represents whether individual i uses the mobile device to use the Internet, Z_i_ represents a series of control variables, province represents the province dummy variable, and ε represents the random disturbance term.

Second, We used Instrumental Variable (IV)-2SLS and GMM methods for estimation due to possible endogeneity issues, taking into account issues such as heteroskedasticity.

#### The influence mechanism of mobile Internet use on mental distress

We selected two mediating variables (trust and happiness) to test the influence mechanism. We discuss three forms of the dependent variable mental distress. First, mental distress is represented by continuous values of CES-D8. Second, distress is a 0–1 dummy variable. The variable is set to 1 if CES-D8 exceeds 16 and zero otherwise. Third, health is another 0–1 dummy variable. The variable is set to 1 if CES-D8 is lower than 16 and zero otherwise.

We used bootstrapping methods to test the mediation effect. The bootstrapping test examines whether the 95% confidence interval of β_1_ × λ_2_ includes the number 0.

#### Further research

In further studies, we examined the different purposes of mobile Internet usage. The questionnaire on the Internet part of CFPS in 2020 included whether to play online games (U91), whether to shop online (U92), whether to watch short videos (U93), whether to study online (U94) and whether to use WeChat (U11). Therefore, we set model (2) to further consider the impact of mobile Internet use for these five different purposes on mental distress.


(2) $$\begin{array}{l} \text{Menta}{\text{l}}_{\text{i}}=\alpha_{0}+\alpha_{1}\text{Mobil}{\text{e}}_{\text{i}}*Purpose_{i}+\alpha_{2}Z_{\text{i}}\\ +\alpha_{3}province+\varepsilon \end{array}$$

Where Purpose_i_ represents the mobile Internet use based on five different purposes (game, shop, video, study, and chat) of individual i.

Moreover, we matched the CFPS data from 2018 with the CFPS data from 2020, setting model (3) and model (4), using probit and logit regression methods.


(3) $$\begin{array}{l} \text{Alleviat}{\text{e}}_{\text{i}}=\alpha_{0}+\alpha_{1}\text{Mobil}{\text{e}}_{\text{i}}+\alpha_{2}Z_{\text{i}}+\alpha_{3}province+\varepsilon \end{array}$$

Where Alleviate_i_ is a 0–1 variable, 1 represents a reduction in mental distress in 2020 relative to 2018, and 0 for others.


(4) $$\begin{array}{l} \text{Alleviat}{\text{e}}_{\text{i}}=\alpha_{0}+\alpha_{1}\text{Adde}{\text{d}}_{\text{i}}+\alpha_{2}Z_{\text{i}}+\alpha_{3}province+\varepsilon \end{array}$$

Where Added_i_ is a 0–1 variable, 1 indicates that an individual is not using mobile online in 2018 but is using mobile online in 2020, and 0 indicates the other situation.

All the above data collation and estimation were done using STATA 16.0.

## Results

### OLS regression

Table 2 shows the results of the estimation using OLS.

**Table 2:** Result of basic OLS regression.

**Variables**	**Mental distress** **CES-D8**	**Mental distress** **CES-D8**	**Mental distress** **CES-D20**	**Mental distress** **CES-D20**
Mobile Internet use	−0.773^***^	−0.459^***^	−1.549^***^	−0.914^***^
	(0.069)	(0.088)	(0.138)	(0.175)
Age	–	−0.004	–	−0.007
		(0.003)		(0.006)
Gender	–	−0.766^***^	–	−1.524^***^
		(0.066)		(0.130)
Marital status	–	−0.531^***^	–	−1.045^***^
		(0.096)		(0.191)
Employment	–	0.422^***^	–	0.834^***^
		(0.078)		(0.155)
Education	–	−0.221^***^	–	−0.439^***^
		(0.021)		(0.042)
Insurance	–	−0.414^***^	–	−0.822^***^
		(0.109)		(0.217)
Constant	12.847^***^	14.871^***^	31.799^***^	35.777^***^
	(0.431)	(0.482)	(0.848)	(0.953)
Province	Yes	Yes	Yes	Yes
Observations	16,004	16,004	16,004	16,004
R-squared	0.030	0.051	0.030	0.051

Robust standard errors in parentheses.

*** *p* < 0.01.

Mobile Internet use relieved 0.773 units of personal anxiety if the control variables and the province dummy variable were not controlled. Mobile Internet use relieved 0.459 units of personal anxiety after controlling for the control variables and the province dummy variable. Both of these results are significant at the 1% level. We used CES-D20 as an alternative variable for regression testing, and the results were still robust. H1 is verified.

### IV method

In order to resolve the potential endogeneity problem associated with traditional OLS, we used an instrumental variable approach to estimate the model (1). We selected two instrumental variables, Mobile Penetration and County average. The two variables are both theoretically macroscopic and are not correlated with other disturbances, such as the personality of an individual.

Table 3 shows the results of the estimation using OLS, 2SLS, and GMM, respectively. It indicates that mobile Internet use can significantly alleviate the mental distress of Chinese adults during COVID-19. However, the impact coefficients differ. 2SLS and GMM have regression coefficients close to 3.014 and 3.034, respectively. Both are greater than the OLS regression coefficients of 0.459.

**Table 3:** Result of instrument variable regressions.

**Variables**	**OLS**	**2SLS**	**GMM**
Mobile Internet use	−0.459^***^	−3.014^***^	−3.034^***^
	(0.088)	(0.600)	(0.600)
Age	−0.004	−0.042^***^	−0.042^***^
	(0.003)	(0.009)	(0.009)
Gender	−0.766^***^	−0.713^***^	−0.713^***^
	(0.066)	(0.068)	(0.068)
Marital status	−0.531^***^	−0.342^***^	−0.341^***^
	(0.096)	(0.107)	(0.107)
Employment	0.422^***^	0.382^***^	0.381^***^
	(0.078)	(0.080)	(0.080)
Education	−0.221^***^	−0.069	−0.068
	(0.021)	(0.042)	(0.042)
Insurance	−0.414^***^	−0.277^**^	−0.274^**^
	(0.109)	(0.117)	(0.117)
Constant	14.871^***^	17.896^***^	17.918^***^
	(0.482)	(0.858)	(0.858)
Province	Yes	Yes	Yes
Observations	16,004	16,004	16,004
R-squared	0.051	–	–

Robust standard errors in parentheses.

*** *p* < 0.01,

** *p* < 0.05.

We conducted a series of tests to verify whether the instrument variables were selected reasonably. The result of the under-identification test showed the *p*-value of the Kleibergen-Paap rk LM statistic <0.01, indicating there is no unidentifiable instrumental variable. Wald F statistics in the weak identification test is >10% maximal IV size, which means there is no existence of weak instrumental variables. The *p*-value of the Hansen J statistic for the over-identification test is 0.234, indicating both instrumental variables selected are exogenous. We also performed the White test, Breusch-Pagan (BP) test, and Weighted Least Square (WLS) to test heteroscedasticity. The results showed that heteroscedasticity existed in the model. Therefore, although the regression coefficients of 2SLS and GMM in Table 4 are similar, the results of GMM are more effective in the presence of heteroscedasticity.

**Table 4:** Regression results by age group.

**Variables**	**Age,** **≥80**	**Age,** **70–80**	**Age,** **60–70**	**Age,** **50–60**	**Age,** **40–50**	**Age,** **30–40**	**Age,** **18–30**
Mobile Internet use	−1.482	−0.575^*^	−0.662^***^	−0.443^***^	−0.517^***^	−0.748^***^	−0.531
	(1.051)	(0.317)	(0.207)	(0.157)	(0.188)	(0.268)	(0.366)
Controls	Yes	Yes	Yes	Yes	Yes	Yes	Yes
Province	Yes	Yes	Yes	Yes	Yes	Yes	Yes
Observations	201	1,271	2,509	3,666	2,834	3,034	2,489
R-squared	0.161	0.117	0.104	0.081	0.070	0.036	0.057

Robust standard errors in parentheses.

*** *p* < 0.01,

^**^p < 0.05,

* *p* < 0.1.

### Heterogeneity analysis

Mobile Internet habits and mental distress levels may differ among different groups. The sample is divided into different groups based on their age, educational level, and whether they live in Hubei provinces.

The sample in this study consisted of individuals aged 18–95. Table 4 indicates that mobile Internet can reduce anxiety and depression in individuals. However, the effect of mobile Internet use on mental distress was not significant among individuals in the age groups 18–30 and 80–95.

As shown in Table 5, mobile Internet use by individuals without a bachelor's degree can significantly relieve mental distress. The Hubei Province was the first to discover COVID-19 in China. And it was also the most severe epidemic area in the country in 2020 (20). Table 6 shows mobile Internet use has increased the mental distress of people in the region.

**Table 5:** Regression by education grouping.

**Variables**	**Bachelor's degree or above**	**Others**
Mobile Internet use	−3.504	−3.062^***^
	(4.514)	(0.656)
Controls	Yes	Yes
Province	Yes	Yes
Observations	1,128	14,876

Robust standard errors in parentheses.

*** *p* < 0.01.

**Table 6:** Regression by province grouping.

**Variables**	**Hubei**	**Others**
Mobile Internet use	5.300^***^	−3.055^***^
	(1.842)	(0.603)
Controls	Yes	Yes
Observations	135	15,869

Robust standard errors in parentheses.

*** *p* < 0.01.

### Mediation analysis

According to Table 7, mobile Internet use can significantly alleviate Chinese adults' mental distress by enhancing their trust or happiness. H2 and H3 are verified.

**Table 7:** Result of media effect.

	**Observed coefficient**	**Bootstrap std. err**.	**95% conf. interval**	
			**Lower**	**Upper**
Mobile → trust → mental distress	−0.037^***^	0.013	−0.062	−0.012
Mobile → mental	−0.416^***^	0.091	−0.595	−0.238
Mobile → happiness → mental distress	−0.070^**^	0.032	−0.132	−0.009
Mobile → mental	−0.385^***^	0.084	−0.549	−0.221
Mobile → trust → distress	−0.003^***^	0.001	−0.006	−0.001
Mobile → distress	−0.033^***^	0.010	−0.052	−0.013
Mobile → happiness → distress	−0.007^**^	0.003	−0.013	−0.001
Mobile → distress	−0.030^***^	0.009	−0.047	−0.013
Mobile → trust → health	0.003^***^	0.001	0.001	0.006
Mobile → health	0.033^***^	0.010	0.014	0.052
Mobile → happiness → health	0.007^**^	0.003	0.001	0.012
Mobile → health	0.030^***^	0.009	0.013	0.047

*** *p* < 0.01,

** *p* < 0.05.

If the dependent variable mental distress is a continuous value of CES-D8, the mediating effect of the variable trust accounted for 8.9% (0.037/0.416) of the direct effect, and the variable happiness accounted for 18.18% (0.07/0.385) of the direct effect. If the dependent variable is the dummy variable distress, the mediating effect of the trust variable accounted for 9% of the direct effect, and the happiness variable accounted for 23.33% of the direct effect. If the dependent variable is the dummy variable health, the mediating effect of trust and happiness is the same as the value of the distress coefficient, but the sign is just the opposite.

## Further research

### Consider the different purposes of mobile Internet use

Table 8 shows that online games, online short videos, online learning, and online chat can significantly alleviate individual anxiety or depression. The impact coefficient of online chat is the highest, which is −0.461^***^. Contrary to expectations, online shopping cannot significantly alleviate mental distress.

**Table 8:** Result of regressions based on the different purposes of mobile Internet use.

**Variables**	**(1)** **Mental distress**	**(2)** **Mental distress**	**(3)** **Mental distress**	**(4)** **Mental distress**	**(5)** **Mental distress**
Game	−0.154^*^ (0.093)	–	–	–	–
Shop	–	0.018 (0.075)	–	–	–
Video	–	–	−0.233^***^ (0.074)	–	–
Learn	–	–	–	−0.211^**^ (0.091)	–
Chat	–	–	–	–	−0.461^***^ (0.088)
Age	0.002 (0.003)	0.003 (0.003)	−0.001 (0.003)	0.002 (0.003)	−0.004 (0.003)
Gender	−0.759^***^ (0.067)	−0.774^***^ (0.066)	−0.771^***^ (0.066)	−0.777^***^ (0.066)	−0.767^***^ (0.066)
Marital status	−0.585^***^ (0.096)	−0.566^***^ (0.095)	−0.549^***^ (0.096)	−0.584^***^ (0.096)	−0.532^***^ (0.096)
Employment	0.420^***^ (0.078)	0.429^***^ (0.078)	0.430^***^ (0.078)	0.434^***^ (0.078)	0.424^***^ (0.078)
Education	−0.248^***^ (0.020)	−0.250^***^ (0.021)	−0.241^***^ (0.020)	−0.240^***^ (0.021)	−0.221^***^ (0.021)
Insurance	−0.439^***^ (0.109)	−0.440^***^ (0.109)	−0.424^***^ (0.109)	−0.434^***^ (0.109)	−0.414^***^ (0.109)
Constant	14.429^***^ (0.475)	14.311^***^ (0.477)	14.590^***^ (0.478)	14.371^***^ (0.471)	14.871^***^ (0.481)
Province	Yes	Yes	Yes	Yes	Yes
Observations	16,004	16,004	16,004	16,004	16,004
R-squared	0.050	0.050	0.050	0.050	0.051

Robust standard errors in parentheses.

*** *p* < 0.01,

** *p* < 0.05,

* *p* < 0.1.

### Consider the impact of the previous data—Use the 2018 and 2020 CFPS data

Table 9 shows that the left two regression coefficients were significantly positive (*p* < 0.05), and the right two regression coefficients were significantly negative (*p* < 0.01). It indicates that mobile Internet use can significantly alleviate individuals' anxiety or depression in general. However, newly enrolled individuals with mobile Internet access experienced increased anxiety. Perhaps new Internet users during the epidemic were anxious to understand the epidemic-related information, and some false information was disseminated on the Internet which led them to become more anxious.

**Table 9:** Result of regressions based on the different purposes of mobile Internet use.

**Variables**	**Alleviate Probit**	**Alleviate Logit**	**Alleviate Probit**	**Alleviate Logit**
Mobile Internet use	0.070^**^ (0.027)	0.112^**^ (0.044)	–	–
Mobile Internet use added	–	–	−0.331^***^ (0.025)	−0.534^***^ (0.041)
Age	0.006^***^ (0.001)	0.009^***^ (0.001)	0.000 (0.001)	0.000 (0.001)
Gender	−0.041^*^ (0.021)	−0.067^*^ (0.034)	−0.032 (0.021)	−0.053 (0.034)
Marital status	−0.128^***^ (0.029)	−0.206^***^ (0.047)	−0.115^***^ (0.029)	−0.187^***^ (0.046)
Employment	0.049^**^ (0.024)	0.079^**^ (0.040)	0.039 (0.024)	0.062 (0.040)
Education	−0.015^**^ (0.006)	−0.024^**^ (0.010)	0.009 (0.006)	0.014 (0.010)
Insurance	0.112^***^ (0.034)	0.185^***^ (0.056)	0.127^***^ (0.034)	0.207^***^ (0.056)
Constant	−0.592^***^ (0.182)	−0.955^***^ (0.295)	−0.103 (0.183)	−0.163 (0.297)
Province	Yes	Yes	Yes	Yes
Observations	16,004	16,004	16,004	16,004
Pseudo R-squared	0.00894	0.00895	0.0165	0.0165

Robust standard errors in parentheses.

*** *p* < 0.01,

** *p* < 0.05,

* *p* < 0.1.

## Discussion

This paper focuses on the effect of mobile Internet use on mental distress among Chinese adults during COVID-19. It shows that mobile Internet use significantly reduces mental distress in 2020. The regression coefficients remained significant after 2SLS and GMM tests using the instrumental variables Mobile Penetration and County average. However, the impact of mobile Internet use on different adults differed. It has a greater effect on Chinese adults aged 30–70, without a bachelor's degree, or living outside Hubei province. According to the mechanism analysis, people can reduce their anxiety or depression by increasing their trust or enhancing their happiness. These conclusions are consistent with those reached by investigations conducted in the United States (37), England (38), or other countries. However, Cotten et al. found that Internet use has a greater impact on the mental health of retired Americans over the age of 50 (39), while we find that the greater impact is on Chinese adults aged 30–70. In addition, Internet addiction is generally defined as spending excess time online, that is, more than 20 h per week (40). Based on CFPS2020 data, only ~0.4% of respondents use mobile Internet for more than 20 h a week. The number of Internet addicts is also predicted by American researchers to be ~ 0.3–0.7% of the total number (41). Thus, Internet addiction is not taken into account in this study.

We also came to other interesting conclusions. The effects of chatting online, playing games, watching short videos, and studying online can alleviate anxiety and depression, with chatting online having the greatest impact coefficient. In contrast to expectations, shopping online does not reduce anxiety or depression among people. It is possible due to regional logistical delays experienced during the epidemic (42). After adding CFPS2018 data to the model for analysis, mobile Internet use still significantly alleviates people's anxiety or depression. However, there was an increase in mental distress among individuals who had recently joined the mobile network in 2020. It is possible that the individuals who joined the mobile network in 2020 primarily went online to find epidemic-related information (43). Searching for this news on the Internet is likely to reinforce negative emotions such as fear (44), which may exacerbate anxiety or depression.

Therefore, this study proposes the following recommendations: Individuals may chat, study, work, and play with mobile internet in order to increase psychological resilience and stress coping ability (31), and reduce their anxiety or depression. Medical institutions may study and implement ways to reduce people's mental distress through the Internet and other means. When preventing and controlling epidemics, governments should take measures to consider the mental distress of specific groups (such as elderly people with less Internet access, people with bachelor's or higher degrees, and areas with more serious epidemics).

This study is of great importance. First, this paper fully discusses the impact of mobile Internet use on people's mental distress during the COVID-19 epidemic, enriching relevant theoretical research. Second, this paper can provide more specific suggestions for using the network to alleviate people's mental distress during the epidemic, which can be used as a reference in the practice. Third, although COVID-19 has been spreading for 2 years, the newest Omicron variant is likely to be more infectious than ever (45). As a result, the study has a theoretical value not only for the short term but also for the long term.

This paper also has some limitations. First, the research should be further refined according to different groups, so that medical institutions and governments can take more targeted measures. Second, this paper uses the CFPS data because of its extensiveness, continuity, and accuracy. The data included some indicators related to the epidemic and Internet use in 2020, but the indicators are not sufficiently refined. In future studies, combining CFPS with special surveys can enhance the accuracy of the research results. Third, we may study the threshold at which mobile networks are capable of alleviating mental distress in the future.

## Conclusions

Mobile Internet use significantly reduced mental distress among adults during COVID-19 in 2020. This conclusion has been strengthened after 2SLS and GMM tests. The extent of the impact of mobile Internet use among different adults showed significant heterogeneity. Adults aged 30–70, without a bachelor's degree or living outside Hubei Province were significantly reduced in mental distress by mobile Internet use, while other groups were less affected. By enhancing trust and happiness, mobile Internet use significantly reduces people's mental distress. Chatting online also reduces mental distress by bringing people closer together. However, there was an increase in mental distress among individuals who had recently joined the mobile network in 2020. Future research can be further refined and deepened based on this study.

## Data availability statement

Publicly available datasets were analyzed in this study. This data can be found here: http://www.isss.pku.edu.cn/cfps/.

## Author contributions

MT contributed to the conception and design of the study, performed the statistical analysis, and wrote the submitted manuscript.

## Funding

This research was funded by the Science and Technology Planning Project of Guangdong Province, China, grant number 2020A1414040040.

## Conflict of interest

The author declares that the research was conducted in the absence of any commercial or financial relationships that could be construed as a potential conflict of interest.

## Publisher's note

All claims expressed in this article are solely those of the authors and do not necessarily represent those of their affiliated organizations, or those of the publisher, the editors and the reviewers. Any product that may be evaluated in this article, or claim that may be made by its manufacturer, is not guaranteed or endorsed by the publisher.
